# SARS-CoV-2 isolation from the first reported patients in Brazil and establishment of a coordinated task network

**DOI:** 10.1590/0074-02760200342

**Published:** 2020-10-23

**Authors:** Danielle Bastos Araujo, Rafael Rahal Guaragna Machado, Deyvid Emanuel Amgarten, Fernanda de Mello Malta, Gabriel Guarany de Araujo, Cairo Oliveira Monteiro, Erika Donizetti Candido, Camila Pereira Soares, Fernando Gatti de Menezes, Ana Carolina Cornachioni Pires, Rúbia Anita Ferraz Santana, Amanda de Oliveira Viana, Erick Dorlass, Luciano Thomazelli, Luis Carlos de Sousa Ferreira, Viviane Fongaro Botosso, Cristiane Rodrigues Guzzo Carvalho, Danielle Bruna Leal Oliveira, João Renato Rebello Pinho, Edison Luiz Durigon

**Affiliations:** 1 Universidade de São Paulo, Instituto de Ciências Biomédicas, Departamento de Microbiologia, São Paulo, SP, Brasil; 2 Hospital Israelita Albert Einstein, São Paulo, SP, Brasil; 3 Instituto Butantã, Centro de Inovação e Desenvolvimento, Laboratório de Virologia, São Paulo, SP, Brasil; 4 Universidade de São Paulo, Faculdade de Medicina, Departamento de Patologia, Laboratório de Medicina Laboratorial (LIM/03), São Paulo, SP, Brasil; 5 Universidade de São Paulo, Faculdade de Medicina, Departamento de Gastroenterologia, Laboratório de Gastroenterologia Clínica e Experimental (LIM/07), São Paulo, SP, Brasil; 6 Plataforma Científica Pasteur-USP, São Paulo, SP, Brasil

**Keywords:** SARS-CoV-2, COVID-19, virus isolation, cell culture, virus network

## Abstract

**BACKGROUND:**

Severe acute respiratory syndrome coronavirus 2 (SARS-CoV-2) was confirmed in Brazil in February 2020, the first cases were followed by an increase in the number of cases throughout the country, resulting in an important public health crisis that requires fast and coordinated responses.

**OBJECTIVES:**

The objective of this work is to describe the isolation and propagation properties of SARS-CoV-2 isolates from the first confirmed cases of coronavirus disease 2019 (COVID-19) in Brazil.

**METHODS:**

After diagnosis in patients that returned from Italy to the São Paulo city in late February by RT-PCR, SARS-CoV-2 isolates were obtained in cell cultures and characterised by full genome sequencing, electron microscopy and *in vitro* replication properties.

**FINDINGS:**

The virus isolate was recovered from nasopharyngeal specimen, propagated in Vero cells (E6, CCL-81 and hSLAM), with clear cytopathic effects, and characterised by full genome sequencing, electron microscopy and *in vitro* replication properties. Virus stocks - viable (titre 2.11 × 10^6^ TCID50/mL, titre 1.5 × 10^6^ PFUs/mL) and inactivated from isolate SARS.CoV2/SP02.2020.HIAE.Br were prepared and set available to the public health authorities and the scientific community in Brazil and abroad.

**MAIN CONCLUSION:**

We believe that the protocols for virus growth and studies here described and the distribution initiative may constitute a viable model for other developing countries, not only to help a rapid effective pandemic response, but also to facilitate and support basic scientific research.

Coronaviruses (CoVs) are single-stranded positive sense RNA viruses that belong to the *Coronaviridae* family. In humans, there were four known endemic CoVs (229E, OC43, NL63 and HKU1) that generally cause mild upper respiratory tract disease with low mortality rates.^1^ However, in 2003 and 2012, respectively, the emergence of highly pathogenic severe acute respiratory syndrome (SARS-CoV)^2^ and Middle East respiratory syndrome (MERS-CoV)^3^ revealed that this virus group may also cause severe respiratory illness in humans. In December 2019, in Wuhan, China, a novel coronavirus, member of the β coronavirus family, has been identified as the source of a pneumonia outbreak^4^ and this novel virus was named as severe acute respiratory syndrome coronavirus 2 (SARS-CoV-2), by the International Committee on Taxonomy of Viruses (ICTV).^5^

In Brazil, the four endemic CoVs circulate annually^6^ ^-^ ^11^ and the first case of coronavirus disease 2019 (COVID-19) was reported on February 26, 2020 (https://covid.saude.gov.br) when SARS-CoV-2 was detected in a 61-year-old male traveller from Lombardia region, Italy, that returned to the São Paulo city, Brazil. Until the first reported case in Brazil, also the first in South American region,^12^ there were 81,109 confirmed COVID-19 cases in 38 countries. After these first reported patient, cases in Brazil started to rise reaching 3,846,153 infected persons and 120,462 deaths on August 29 (https://covid.saude.gov.br). 

Isolation and propagation of new viruses *in vitro* represents an essential step and may generate important primary tools in early outbreak characterisation. In that way, isolation of the virus presently disseminating in Brazil will provide important information regarding diversity and molecular evolution of the pathogen, but also supply reference material in the struggle to control the rampant spreading of the pandemic SARS-CoV-2 in the country. Indeed, the availability of infective particles as well as inactivated genetic material as reference reagents are extremely necessary for the preparation of positive controls in molecular diagnosis, development of vaccine formulations, detection of neutralising antibodies, screening of antiviral compounds and for different basic research projects both for public health reference laboratories and the research community. 

In this study, we describe the isolation of SARS-CoV-2 from the first two patients diagnosed with the novel coronavirus disease (COVID-19) in Brazil. We describe its genomic sequence (SARS-CoV-2/SP02/human/2020/BRA) and *in vitro* replication characteristics. Virus stocks (infectious particles and lysates) were set available and distributed to the research community.

## MATERIALS AND METHODS

*Ethics declarations -* All methods were performed in accordance with relevant guidelines and regulations. This work was approved by the Ethics Committee on Research with Humans from the Institute of Biomedical Sciences, University of São Paulo, Brazil (permission number 74683917.1.0000.5467). All specimens were handled under the Laboratory biosafety guidance required for the novel coronavirus (2019-nCoV) by the World Health Organization (WHO)^13^ at BLS3 facilities at the Institute of Biomedical Sciences, University of São Paulo.

*Clinical specimen collection -* Nasopharyngeal (NP) swab samples were collected from symptomatic patients who had acquired COVID-19 during travels to northwest of Italy (Lombardia region) and returned to the São Paulo city in late February. These patients were treated in the same hospital and were the two first confirmed cases of COVID-19 in the São Paulo city. The specimens were collected on day 2-4 post-symptom onset, placed in 1-2 mL of saline medium and used for molecular diagnosis and virus isolation.

*Nucleic acid extraction and real-time RT-qPCR for virus detection -* In order to perform the identification of SARS-CoV-2, the extraction of total nucleic acid (RNA and DNA) from the collected samples (200 µL of initial material) were carried out using the semi-automated NucliSENS^®^ easyMag^®^ platform (bioMérieux, Lyon, France), following the manufacturer’s’ instructions. All specimens were handled under the laboratory biosafety guidance required for the novel coronavirus (2019-nCoV) by WHO^13^ at BLS3 facilities at the Institute of Biomedical Sciences, University of São Paulo. The detection of viral RNA was carried out using the AgPath-ID One-Step RT-PCR Kit (Applied Biosystems Inc., Waltham, USA) on an ABI 7500 SDS real-time PCR machine (Applied Biosystems, Weiterstadt, Germany), using a published protocol and sequence of primers and probe for E gene.^14^ RNA copies/mL was quantified by real-time RT-qPCR using a specific *in vitro*-transcribed RNA quantification standard, kindly granted by Christian Drosten, Charité - Universitätsmedizin Berlin, Germany, as described previously.^2^

*Virus isolation -* We used Vero E6 cells for isolation and initial passages. We cultured Vero E6 in Dulbecco minimal essential medium (DMEM) supplemented with 10% of heat-inactivated foetal bovine serum (FBS) (Vitrocell Embriolife, Campinas, Brazil). 

We used NP swab specimen for virus isolation. For isolation and first passage, we sow cells in a 25 cm^2^ cell culture flask in a concentration of 5 × 10^5^ cells/mL. After 24 h, we removed the culture medium, washed three times with FBS free-DMEM and inoculated aliquots (500 μL) of the clinical specimens into the flask. After 1 h of incubation (adsorption), we completed the volume for 5 mL with DMEM supplemented with 2.5% FBS and 1% of penicillin-streptomycin. We grew the inoculated cultures in a humidified 37°C incubator in an atmosphere of 5% CO_2_ and observed for cytopathic effects (CPE) daily up to 72 h. Supernatant was collected daily, and virus replication was confirmed through CPE, gene detection and electron microscopy.

*Virus titration -* Median tissue culture infectious dose (TCID_50_/mL) - Vero E6 and CCL-81 cells were seeded into 96-well plate (5 × 10^4^ cells/mL), 24 h before the experiment. Virus was 10-fold serially diluted in medium (10^-1^ to 10^-12^). Medium was removed from plates, virus dilutions applied in sextuplicate and incubated at 37°C. Visualisations were performed daily in an inverted light microscope (Axiovert 100, Carl Zeiss Oberkochen) to observe the CPE. After 72 h, the last reading was performed, and the monolayers were fixed and stained with Naphthol Blue Black (Sigma-Aldrich Co., Deisenhofen, Germany) dissolved in sodium acetate-acid acetic. The viral titre was expressed in TCID_50_/mL and calculated using the Spearman & Kärber algorithm, as described by Hierholzer & Killington.^15^

Plaque forming units (PFU/mL) - Virus titration was carried out in 24 wells plates seeded with Vero E6 and CCL-81 cells at a concentration of 1 × 10^5^ cells/well. After 24 h and a cell confluence of 80-90%, dilutions 10^-1^ to 10^-10^ in DMEM 2.5% FBS of the virus was transferred in duplicate (100 µL/well) to the seeded plates. After 1 h adsorption at 37^o^C 5% CO_2_, the wells were completed with an overlay of carboxymethyl cellulose (CMC) with DMEM, 2% FBS and 1% of penicillin-streptomycin, and plates incubated at 37^o^C in 5% CO_2_ and stained with Naphtol Blue Black dissolved in sodium acetate-acid acetic. Plates were observed and stained from 48 to 96 h post-inoculation (h.p.i.). Both virus titration (TCID_50_/mL and PFU/mL) were made after the third passage of the isolated virus (T2).

*Negative stain transmission electron microscopy -* Samples were adsorbed to glow-discharged carbon film-coated copper grids (400 Mesh, CF400-Cu, Electron Microscopy Sciences). The grids were washed with ultrapure water treated with DEPC and negatively stained with uranyl acetate 2% (w/v) with blotting on filter paper after each step. A FEI Tecnai G20 200 kV transmission electron microscope (Department of Cell and Developmental Biology, Institute of Biomedical Sciences, University of São Paulo) was used for image acquisition.

*Virus growth kinetics in different cell lines -* Three Vero cell lines (E6, CCL-81 and hSLAM) plus a human epithelial type 2 (HEp-2) cells, at concentration of 5 × 10^4^ cells/mL, were tested for the propagation of the SARS-CoV-2 by inoculation at a multiplicity of infection (MOI) of 0.02. The culture medium consisted of DMEM supplemented with 2.5% of FBS. Aliquots of cell-associated and supernatants compartments were collected every 12 h up to 96 h.p.i. for virus quantification via TCID_50_/mL and RNA copy number quantification by reverse transcription-quantitative polymerase chain reaction (RT-qPCR). The assay was conducted in triplicate, reproduced in two independent experiments and expressed by standard error of the mean (SEM). Graphics and SEM were done using GraphPad Prism software version 8.1 (GraphPad Software, San Diego, USA).

*Next generation sequencing of viral full-length genome -* We extracted total nucleic acid from the NP and oropharyngeal (OP) swab samples and cell supernatants isolates with the QIAamp Viral RNA Mini kit (QIAGEN, Hilden, Germany). The purification and concentration steps were carried out with RNA Clean & Concentrator kit (Zymo Research, Irvine, USA) with DNAse I treatment during the concentration process. Depletion of human ribosomal RNA was performed with the concentrated RNA product using the QIAseq Fast Select RNA Removal kit (QIAGEN). Finally, the RNA samples were submitted to random amplification following the methodology described in Greninger et al.^16^ with few modifications.

The preparation of sequencing libraries for the Illumina platform was carried out with the Nextera XT Kit (Illumina, San Diego, USA) and multiplex testing, using the random two-step PCR amplification product as input, followed the kit’s standard instructions. The libraries were quantified after fluorescence measuring with the Qubit instrument (Thermo Fisher Scientific, Waltham, USA) and loaded on the NextSeq 550 equipment (Illumina) for sequencing with MID 300 paired-end reads (Illumina).

*Sequencing analysis -* The sequencing data was analysed by a flow of bioinformatics analysis (pipeline) developed at Albert Einstein Hospital. In summary, raw sequencing data was subjected to sequence quality controls, removal of human contaminants by aligning against the HG19 reference genome, taxonomic identification of other pathogens and genome recovery through manual curing. Quality control was performed using cutadapt^17^ to filter sequences by length (< 50 bp), average quality (Q_p_ < 20) and trim options to remove low quality ends (9 bp to 5’ end and 5 bp to 3’ end). Passed QC reads were mapped to HG19 human reference genome using bwa^18^ mem with default parameters. Not mapped reads were submitted to assembly using SPADES 1.13.^19^ Contigs were inspected and manually curated using Geneious 2020.1 to generate a final assembly. Complete genome was compared to SARS-CoV-2 reference and close isolates by multiple sequence alignment. Final genome was deposited in GenBank (https://www.ncbi.nlm.nih.gov/genbank/).

*Production of virus isolates (VIS) and lysate (VLS) stocks and national distribution network -* The preparation of VIS and VLS stocks was performed as described above for virus isolation. Related to VIS, one millilitre of VIS (passage 3, T2) was aliquoted on cryogenic vials (Corning Incorporated, Kennebunk, USA) and stored at -196°C. For VLS, 200 µL of T2 of SARS-CoV-2 isolate was added to 800 µL of NucliSENS^®^ Lysis Buffer (bioMérieux) for virus inactivation and conservation of genetic material in cryogenic vials (Corning Incorporated) and stored at -20°C. The delivery of VIS and VLS was made by the Brazilian Mail Company in partnership with Ministry of Science, Technology and Innovations (MCTIC) of Brazil (http://www.mctic.gov.br) in accordance with the Brazilian Health Regulatory Agency (ANVISA) biosafety rules. Upon formal request to acquire VIS and/or VLS, all recipients signed a term of commitment and responsibility for the use of such reagents within the norms stipulated by WHO.^13^

## RESULTS AND DISCUSSION

*Clinical specimen collection -* Patient 1 (HIAE01), a 61-years-old male patient, and patient 2 (HIAE02), a 32-years-old male patient, had returned from northwest of Italy (Lombardy region) and presented respiratory symptoms including cough, sore throat, runny nose, fever, myalgia and headache. Patient 1 was initially diagnosed with a community-acquired pneumonia and received antimicrobial therapy. Both were confirmed for COVID-19 by Hospital Israelita Albert Einstein (HIAE), in the São Paulo city on February 26 (HIAE01) and 28 (HIAE02), 2020. A summary of clinical characteristics of the patients is described in Table. Lombardy is considered the centre of the COVID-19 outbreak in Italy^20^ and has a high influence of the first wave of SARS-CoV-2 introduced in Brazil.^21^

**TABLE:** Clinical characteristics of the two patients (HIAE01 and HIAE02) reported as the first two coronavirus disease 2019 (COVID-19) cases in Brazil

	HIAE01	HIAE02
Age (years)	61	32
Sex	Male	Male
Symptoms	Fever, cough, sore throat, runny nose	Cough, sore throat, fever, myalgia and headache
Air travel return to the São Paulo city	February 21	February 27
Onset of symptoms	February 23	February 28
Sample collection date	February 25	February 28
Diagnosis date	February 26	February 28
Travel history	Northwest Italy (Lombardy region)	Northwest Italy (Lombardy region) and Switzerland (Saint Moritz city)

HIAE: Hospital Israelita Albert Einstein.

*Virus isolation -* Before isolation in cell cultures, we tested the samples using a one-step multiplex RT-qPCR for the detection of 18 additional different respiratory viruses^22^ and tested for bacterial contamination using Fluid Thioglycolate Medium (Becton Dickinson, Franklin Lakes, USA). No other pathogens were detected.

**Fig. 1:**
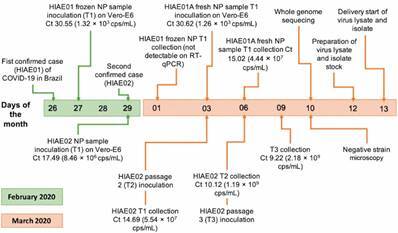
schematic timeline of virus isolation study of the first two confirmed cases of coronavirus disease 2019 (COVID-19) in Brazil. Cps: copies; Ct: cycle threshold; HIAE: Hospital Israelita Albert Einstein; NP: nasopharyngeal; RT-qPCR: quantitative reverse transcription polymerase chain reaction.

The positive NP were inoculated on Vero E6 cells. The initial sample collected from HIAE01 was freezed and thawed before inoculation and no virus propagation was obtained. A second sample, from the same patient, was obtained “fresh” (conserved at 4ºC for no longer than 12 h) and we could successfully isolate SARS-CoV-2. Sample from HIAE02 was inoculated “fresh” from the first moment. The failure to isolate the virus from the first sample collected from HIAE01 may be attributed to a lower virus load and to the freeze-thaw cycle before cell culture inoculation. The schematic timeline of procedures is presented at Fig. 1.

**Fig. 2:**
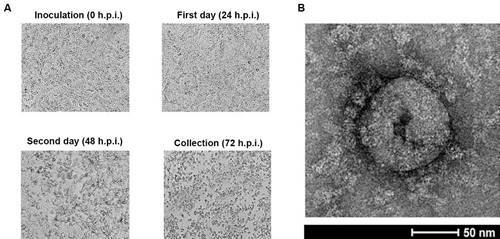
microscopy of severe acute respiratory syndrome coronavirus 2 (SARS-CoV-2) isolate in Brazil. (A) Optical microscopy of Vero E6 cell line, up to 72 h post-inoculation (h.p.i.), showing the cytopathic effects caused by SARS-CoV-2 from a nasopharyngeal swab sample from a patient (HIAE02) with coronavirus disease 2019 (COVID-19), Brazil, 2020. Original magnifications 100X. (B) Negative stain transmission electron microscopy of the SARS-Cov-2 isolate using uranyl acetate 2% at a magnification of 100,000X. Bars: 50 nm.

Three days post infection, the isolation of SARS-CoV-2 from HIAE02 was confirmed by RT-qPCR, electron microscopy and whole genome sequencing (Fig. 1). The cycle threshold (Ct) value and genome copy numbers (RNA copies/mL) of the pre-inoculated sample was Ct 17.49 and 8.46 × 10^6^ copies/mL, respectively, showing lower viral loads when compared to the post-inoculated supernatant samples [16.93 - 1.23 × 10^7^ (24 h.p.i.), 14.69 - 5.54 × 10^7^ (48 h.p.i.) and 10.82 - 7.44 × 10^8^ (72 h.p.i.), respectively]. Vero E6 cells exhibited clear CPE at 48 h.p.i., being harvested at 72 h.p.i. (Fig. 2A). RNA quantification of passages 2 and 3 after 72 h.p.i. gave values of 10.12 - 1.19 × 10^9^ copies/mL and 9.22 - 2.18 × 10^9^ copies/mL, respectively. Since virus isolation from HIAE02 (SARS.CoV2/SP02.2020.HIAE.Br) was faster, all the subsequent studies were carried out with this isolate after passage 3. Virus isolation (passage 0) from HIAE01 (SARS.CoV2/SP01.2020.HIAE.Br) was confirmed by RT-qPCR (Ct 15.02 - 4.4 × 10^7^ copies/mL) and whole genome sequencing, being stored at -80ºC. 

Negative staining transmission electron microscopy of the SARS.CoV2/SP02.2020.HIAE.Br, here after referred as SP02/BRA, permitted the observation of coronavirus-specific morphological structure, being possible to visualise the protein components of the viral envelope. The virus particle size ranged from 80 to 100 nm (Fig. 2B). 

*CPE and virus titration -* The CPEs were characteristic of SARS-CoV-2: cell rounding, detachment of the cell monolayer and formation of loose cells on the surface, for both Vero E6 and CCL-81 cells and similar to previously observed effects.^23^ ^-^ ^26^ Nonetheless, the CPEs were more evident in CCL-81 cells. The virus isolate was titrated after two blind passages following isolation (T2) and the CPEs were more clearly observed in CCL-81 cells for TCID_50_/mL (2.11 × 10^6^) and PFUs (1.5 × 10^6^). For PFUs titration, effects were not clear for Vero E6 cells, and for Vero CCL81, only a few small plaques were visible at 72 h.p.i., being much larger and more visible at 96 h.p.i. (Supplementary data, Fig. 1). Hartcourt et al.^23^ described effects more visible for Vero E6 cells when compared to CCL-81. Other successful SARS-CoV-2 isolation reports were based on Vero E6, Vero CCL-81 and Vero hSLAM^23^ ^-^ ^27^ showing that the three cell lines are permissive for SARS-COV-2.

**Fig. 3:**
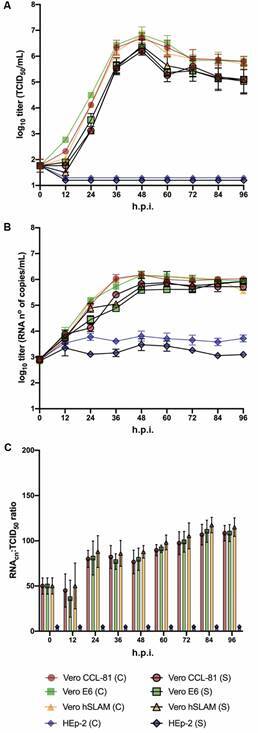
replication kinetics of severe acute respiratory syndrome coronavirus 2 (SARS-CoV-2) in four different cell lines (Vero E6, Vero CCL-8, Vero hSLAM and HEp-2). Cells were inoculated at a multiplicity of infection (MOI) of 0.02. Virus from the different cell (C) and supernatant (S) were harvested and acquired every 12 h during 96 h post-inoculation (h.p.i.) and quantified by either tissue culture infectious dose (TCID_50_/mL) and RT-qPCR. Time courses were determined in triplicates, by two independent experiments and the error bars represent standard error of the mean (SEM). (A) Time course quantification of infectious virus titres are indicated the tested cell lines. (B) Quantification of virus RNA copy numbers by RT-qPCR. (C) RNA_cn_:TCID_50_ ratios. RNA_cn_: ribonucleic acid copy number.

*Virus growth kinetics in different cell lines -* We examined SARS-CoV-2 growth kinetics in three Vero cell lines (E6, CCL-81 and hSLAM) and compared with HEp-2 cells. All cells were inoculated with the SARS-CoV-2 isolate (SP02/BRA) and cultured under similar conditions. Virus titre quantification analysis of cell-associated and supernatants compartments indicated that similar levels of infectious SARS-CoV-2 were produced in all three Vero cell lines of Vero, but not in HEp-2 cells, that proved to be not permissive to virus replication. The peak of viral titre was detected 48 h.p.i. (10^7^ TCID_50_/mL) after an initial eclipse phase. CPEs was not observed until 48 h.p.i. and reached a peak at 72 h.p.i. (Supplementary data, Fig. 2). Quantification of ribonucleic acid copy number (RNA_cn_) indicated that virus was released into the supernatant with similar kinetics for all three tested Vero cell lines, with virus yielding slightly higher values on 72 h.p.i. (Fig. 3B). RNAcp:TCID_50_ ratios did not differ significantly (p > 0.05) among the tested Vero cell lines (Fig. 3C). In addition, these cell lines appeared to release few noninfectious particles at early time points of the infection. The RNAcp:TCID_50_ ratios appeared to increase discreetly over time, suggesting an increase in the release of noninfectious virus at later time points or an increase in virus particle degradation over time (perhaps as a consequence of cell culture proteases). Virus RNA was not detected in the cell-associated fraction and cell cultures supernatants of HEp-2 cells (Fig. 3A, B). Any clear-characteristic CPE of SARS-CoV-2 was observed in HEp-2 cells (Supplementary data, Fig. 2). Similarly to other studies with SARS-CoV^28^ ^,^ ^29^ and SARS-CoV-2,^23^ ^,^ ^26^ our findings demonstrated that the three tested Vero cell lines release infectious virus particles and viral RNA copies at the same kinetics and efficient egress.

**Fig. 4:**
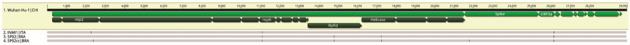
multiple sequence alignment among Wuhan-Hu-1 and INMI1/ITA reference sequences and SARS-CoV-2 isolated from an early patient in Brazil (SP02/BRA isolate). The isolate was cultivated in Vero E6 cells and whole genome sequencing was performed in the second passage (SP02cc/BRA isolate). Two non-synonymous single nucleotide polymorphisms (SNPs) were observed in the cultivated isolate, respectively at positions 2,388 bp and 21,784 bp from Wuhan reference. First SNP resulted in the amino acid change T708I in peptide nsp2, while second SNP resulted in the amino acid change N74K in spike protein. SNPs observed in SP02 isolates are shown by a black straight line at the respective sequence. Features by colour: light green represents genes; dark green represents mature peptides; and light grey represents untranslated regions. Gene or protein name associated with sequence changes are given in the genome schematic.

*Whole genome sequencing -* Whole genome sequence of the SARS-CoV-2 Wuhan-Hu-1 (NC_045512) and INMI 1/ITA (MT066156) were compared with SP02/BRA directly isolated from patient’s sample (MT126808) and after cultivation in Vero cells (MT350282) using MAFFT multiple aligner tool (algorithm = auto and PAM = 1). Alignment shows only two mutations at the cultivated strain (> 99.993% similarity). Mutations occurred at the nsp2 and spike proteins (Fig. 4).

**Fig. 5:**
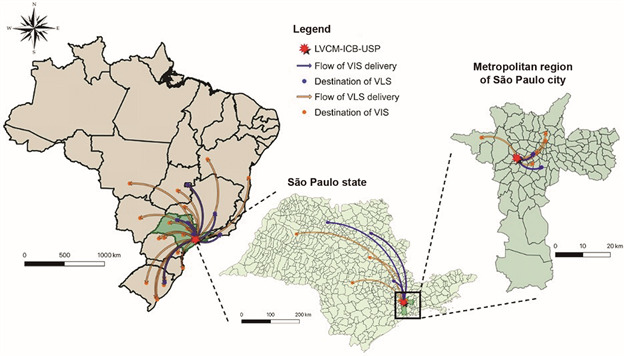
distribution network flow map showing the delivery destinations of SP02/BRA samples virus isolates (VIS) and lysates (VLS) to different research groups in Brazil, by the Laboratory of Clinical and Molecular Virology (LVCM) of the Institute of Biomedical Sciences (ICB), University of Sao Paulo (USP). The image was designed using QGIS 3.6.1 software (http://www.qgis.org/en/ site/about/index.html).

*Distribution network -* Until March 20, 2020, the sending of the material comprised 31 different research groups, in public and particular university/hospitals, at 10 different states in Brazil (Fig. 5). The inactivated virus (VLS) was distributed according of request from the laboratories to testing clinical samples by RT-qPCR, using the VLS as positive controls, which was important, considering the difficulties - availability and price - to import synthetic RNA in Brazil. The criteria for distributing live virus (VIS) was based in the capacity of BSL3 facilities from the host institutions, experience of the principal investigator and the analysis of priority for development of vaccine, drug discovery and virus neutralisation diagnosis. This initiative is crucial to improve the study of SARS-CoV-2 and the development of methods and strategies for virus treatment and prevention. SARS-CoV-2 isolates were set available to the scientific community by different groups in other countries.^23^ ^,^ ^27^ The first delivery to the research community at the São Paulo state was set on March 13. The virus distribution by the Brazilian Mail Company was first set on March 18, 2020 and, in less than 13 h, the biological material was delivered to Rio de Janeiro (222.26 miles away from the São Paulo city), the state of Minas Gerais (304.28 miles) and the state of Rio Grande do Sul (815.94 km). Virus samples were sent following technical and biosafety requirements, in accordance with ANVISA recommendations. 

In conclusion, in this work, we describe the successful isolation of SARS-CoV-2 from the first diagnosed patients in Brazil. The virus was propagated in Vero cell lines and replication features, CPE and growth kinetics were described. The experimental protocols described herein can be used for future attempts to isolate SARS-CoV-2 in different places in the world. The produced virus stocks were distributed to different research groups and hospitals in the country and are being used as a reference in diagnostic tests and for research, aiming the screening of antivirus drugs, testing the efficacy of vaccine formulations under experimental conditions. The VLS are been used as controls for molecular diagnosis and studies, eliminating the need of imported material in the first weeks of the pandemic in Brazil. The COVID-19 pandemic is unprecedent and the collaborative work is crucial to the efforts to understand and control the virus spread in the country.

*Data availability -* The complete genome sequences of SARS-CoV-2/SP02/human/2020/BRA from de clinical sample and SARS-CoV-2/human/BRA/SP02cc/2020 from cell culture isolation have been deposited in the GenBank (accession MT126808 and MT350282, respectively). The version described in this paper is the first version.
