# Supplementary material for: SARS-CoV-2 isolation from the first reported patients in Brazil and establishment of a coordinated task network
Source: Mem Inst Oswaldo Cruz. 2020 Oct 23;115:e200342. doi: 10.1590/0074-02760200342 (PMC7586445; doi:10.1590/0074-02760200342)
Supplement: Supplementary file 1 [file 1678-8060-mioc-115-e200342-s.pdf]

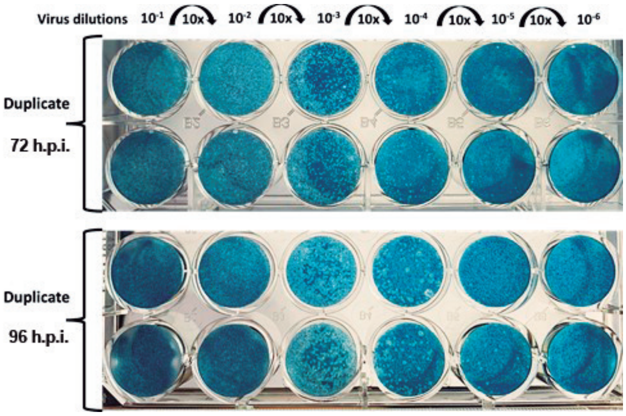

Fig. 1: severe acute respiratory syndrome coronavirus 2 (SARS-CoV-2) titration by plaque forming units (PFUs) in two different time-points [72 and 96 h post-inoculation (h.p.i.)]. Vero CCL-81 cells were infected with 10-fold serial dilutions (10^{-1} to 10^{-6}) of SP02/BRA overlaid and stained with Naphthol Blue Black. The final viral titre was calculated to be 1.5×10^6 PFU/mL.

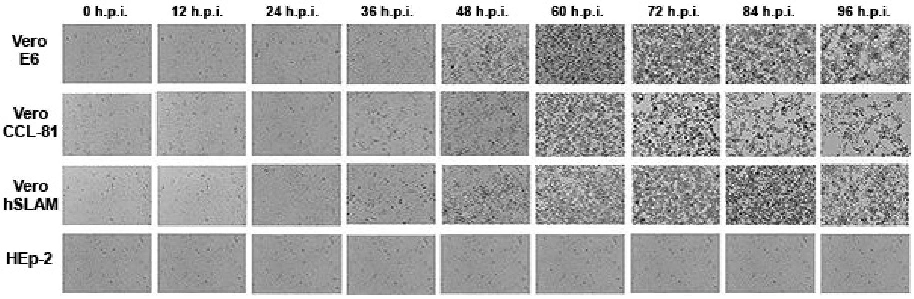

Fig. 2: cytopathic effects (CPEs) visualised by light microscopy in the indicated cell lines (Vero E6, CCL-81, hSLAM and HEp-2) up to 96 h post infection (h.p.i.) with SP02/BRA at a multiplicity of infection (MOI) of 0.02. The presence of CPEs becomes more evident at 48 h.p.i., when we observed 50% of cell lysis. Final magnification: 100X.
